# Fast TWIST with iterative reconstruction improves diagnostic accuracy of AVM of the hand

**DOI:** 10.1038/s41598-020-73331-6

**Published:** 2020-10-01

**Authors:** Veronika I. Huf, Claudia Fellner, Walter A. Wohlgemuth, Christian Stroszczynski, Michaela Schmidt, Christoph Forman, Jens Wetzl, Wibke Uller

**Affiliations:** 1 Department of Radiology, University Medical Center Regensburg, 93042 Regensburg, Germany; 2 Interdisciplinary Center for Vascular Anomalies, University Clinic and Polyclinic of Radiology, University Hospital Halle, 06120 Halle (Saale), Germany; 3 Siemens Healthcare, 91052 Erlangen, Germany

**Keywords:** Medical research, Magnetic resonance imaging

## Abstract

Very high temporal and spatial resolution is mandatory for the diagnosis of arteriovenous malformations (AVM) of the hand. Until now, magnetic resonance imaging (MRI) has not fulfilled both requirements simultaneously. This study presents how the combination of a very fast TWIST MRI (time-resolved angiography with interleaved stochastic trajectories) sequence and iterative reconstructions optimizes temporal as well as spatial resolution. 11 patients were examined at a 3-T MRI scanner with two different TWIST protocols: the standard and the study protocol, acquiring a data set every 5.57 s and 1.44 s respectively. The study data was retrospectively iteratively reconstructed with different regularization factors (0.001, 0.002, 0.004, 0.008). Results were compared using the sign-test. P-values < 0.05 were regarded statistically significant. With a low amount of contrast medium, the temporal resolution of the study protocol enabled the differentiation of arteries from veins in all patients whereas the signal-to-noise ratio (SNR) deteriorated. Depending on the regularization factors, SNR, delineation of arterial feeders and non-involved hand and interdigital arteries, as well as artefact levels varied. Overall, iterative reconstruction with regularization factor 0.004 achieved the best results, consequently showing the ability of MRI as a reliable diagnostic method in AVMs of the hand.

## Introduction

According to Mulliken et al.^1^, vascular anomalies are divided into vascular tumours and vascular malformations. The latter ones occur in approximately 0.3–0.5% of the population^2^. Based on their rheological behaviour, they are subcategorized into high-flow and low-flow malformations^3^. Although representing the most common type of high-flow malformations, peripheral arteriovenous malformations (AVMs) are very rare and occur in 14.3% of patients suffering from vascular malformations^4^. Of these, 28.5% of peripheral AVMs are located on the upper and lower extremities, mostly sparing the feet and hands^4^. AVMs consist of a direct shunting area between arteries and veins. The inner centre of an AVM is the reticulate nidus with fine-spun shunts between tiny arteries and veins. Patients with AVMs may suffer from pain, aesthetical impairment, ulcer, bleeding, and increased cardiac output^5,6^.

Radiological interventional therapy of AVM aims for the complete occlusion of the nidus and is a well-established treatment option^7,8^. However, the occlusion of non-involved arterial vessels that provide the blood supply of tissue distal of the AVM has to be prevented to avoid tissue necrosis or amputation in the worst case^6^. Most importantly, the angioarchitecture and hemodynamics of the AVM have to be fully understood for treatment planning^9^. As the blood flow in AVMs is very fast and diameters of vessels may be very small, the perfect imaging modality for intervention planning needs to combine an extremely high temporal and a high spatial resolution^10,11^.

Both can be achieved with conventional digital subtraction angiography (DSA)^11–13^. The disadvantages of DSA are radiation exposure, iodine-contrast medium injection, and the technique’s inherent invasiveness with the risk of bleeding and vessel disruption^10^. Furthermore, morphologic information about the surrounding tissue is lacking.

A generally accepted imaging alternative is dynamic contrast-enhanced magnetic resonance angiography (MRA)^14^. After the injection of intravenous gadolinium contrast repeated MRA data sets are acquired^15^. For the imaging of high-flow vessels, a high acquisition speed is mandatory. Techniques like TWIST (time-resolved angiography with interleaved stochastic trajectories) MRA, which images k-space centre more frequently than peripheral portions, achieve a high temporal resolution^15^. Contrary to single-phase MRA, TWIST MRA is independent of the perfect contrast bolus timing^16^. However, side effects of these new techniques accelerating the image rate are blurring, ghosting, and a loss of signal-to-noise ratio (SNR), which interferes with the image quality^10^ and masks smaller vessels and their hemodynamics especially in peripheral anatomic regions.

Recent studies revealed that iterative reconstructions seem to be beneficial for MRA by enabling fast sequences and improving the visualization and demarcation of micro vessels^15,17^. AVMs exclusively located on the hand are difficult to image and have never been examined in this setting because of the small size of the feeding and draining vessels and the fact that these are mostly terminal vessels. Accordingly, this study investigated the temporal and spatial resolution of a very fast TWIST study protocol of AVMs involving the hand. Due to the high-flow situation in extremely tiny vessels, the correct depiction of hemodynamics is challenging. Study data sets were retrospectively reconstructed with four different regularization factors and results were compared to our standard TWIST protocol.

Thereby, we examined the value of the study TWIST MRA in improving the temporal resolution in high-flow vessels. Furthermore, we investigated if iterative reconstructions with different regularization factors are able to optimize the study protocol, especially in delineating very small vessels and in diminishing artefacts.

## Methods

Institutional Review Board approval for this study was obtained.

From 02/2016 to 10/2016, 11 consecutive patients who presented at our tertiary care Vascular Anomalies Centre (VAC) with AVMs of the hand or the fingers where included in this study and examined according to a predefined protocol with a 16-channel hand/wrist coil at a 3-T MR system (MAGNETOM Skyra, Siemens Healthcare, Erlangen, Germany)^14^.

MRA was indicated for interventional radiological treatment planning or for follow-up imaging after therapy.

All patients gave their written informed consent for the MRI examination including intravenous application of contrast medium. Contraindications for intravenous contrast medium applications like allergies or kidney disease were ruled out. The cumulative applied dose of contrast medium was 0.1 mL/(kg body weight) per patient (Gadobutrol, Gadovist, Bayer vital, Leverkusen, Germany).

Standard and study protocol were executed during the same examination. Primarily the standard TWIST protocol was performed. For every affected level of the upper extremity, like the upper arm, the forearm, or the hand, a separate contrast medium injection was executed. Afterwards the study protocol with one contrast medium administration for the hand was performed. Thus, the maximal single dose of contrast medium was divided through the number of affected levels for the standard protocol plus one injection for the study protocol, resulting in the available contrast medium amount per injection.

Contrast medium was administered by power injector with a flow of 2 mL/s.

According to the routine protocol using parallel imaging with an acceleration factor of 3 (TWIST_3) in phase-encoding-direction, a 3D data set was reconstructed every 5.57 s with a measured voxel size of 0.77 mm × 0.70 mm × 0.83 mm.

The study TWIST protocol was applied with an acceleration factor of 12 (TWIST_12): 4 (phase-encoding-direction) × 3 (in partition direction). A 3D data set was reconstructed using a prototype iterative approach^15^ each 1.44 s with a measured voxel size of 0.72 mm × 0.72 mm × 0.72 mm. Image calculation was initially performed with the standard view sharing technique. In addition to the product reconstruction, retrospective iterative reconstructions of the same datasets were performed using four different regularization factors (0.001, 0.002, 0.004, 0.008). Thus, five differently reconstructed study protocols were assessed.

Standard TWIST alternates the acquisition of k-space center (A) region and differently undersampled k-space periphery (B) regions, where combining multiple B regions results in a regular undersampling suitable for standard parallel imaging reconstruction, i.e. a sliding window covering one A and multiple B regions. According to Wetzl et al., for iterative reconstruction, consecutive single A – B pairs were reconstructed. Thus, a reduction of the temporal footprint and an increase of undersampling were achieved^15^. To recover the frames $\{\widehat{x}_{t}\}$_*t*=1,…,*T*_ for all time points *T*, a nonlinear, iterative SENSE-type reconstruction with spatio-temporal wavelet regularization was performed:

$$\{ {{\widehat x_{t}}}\}_{{t =1, \ldots ,T}} = argmin_{{\{ x_{t}\} }} \mathop \sum \limits_{t = 1}^{T} \left( {\mathop \sum \limits_{c = 1}^{C} \parallel {\varvec{A}}_{t} {\varvec{FS}}_{c} {\varvec{x}}_{t} - {\varvec{y}}_{t,c} \parallel_{2}^{2} + \lambda_{\sigma } \parallel {\varvec{W}}_{\sigma } {\varvec{x}}_{t} \parallel_{1} } \right) + \lambda_{\tau } \parallel {\varvec{W}}_{\tau } \left( {{\varvec{x}}_{1}^{{\text{T}}} \ldots {\varvec{x}}_{T}^{{\text{T}}} } \right)^{{\text{T}}} \parallel_{1}$$

The abbreviation C represents the number of coils and ***A***_*t*_ the sampling pattern for time *t*. ***F*** is the Fourier transform, ***S***_*c*_ the multiplication by the sensitivity of coil *c*, ***y***_*t,c*_ the measured data for time *t* and coil *c* (from an A and B region), λ_σ_ and λ_τ_ are the spatial and temporal regularization parameters and ***W***_σ_ and ***W***_τ_ are spatial and temporal redundant Haar wavelet transforms ^15^. The temporal and spatial regularization factors were related by a fixed factor λ_τ_ = 5λ_σ_. All regularization factors in the manuscript refer to the spatial factor. The regularization coefficients were scaled by the maximal intensity in each dataset to make the selection of regularization parameters independent of intensity variations across subjects.

Reconstruction was performed at the scanner directly utilizing its graphic processing unit (GPU).

For comparison between TWIST_3, TWIST_12, and TWIST_12 reconstructed with four different regularization factors, the following parameters were analysed on the maximum intensity projections (MIPs):

### Delay

Dependent on the extent of the AVM, a region of interest (ROI) was placed in the radial/ulnar artery and the adjacent dominant outflow vein; time-intensity curves were calculated for each ROI and time lag between arterial and venous peak enhancement was determined. Results between the TWIST_3 standard protocol and the study protocol iteratively reconstructed with the regularization factor 0.004 were compared with each other.

### SNR

Three different ROIs (area: 0.02 cm^2^ each) were placed within a major hand artery on TWIST_12 and all iteratively reconstructed data sets at exactly the same position as soon as the vessel was visible. The mean signal intensity was divided by the standard deviation within these ROIs, thus an apparent “vessel SNR” was calculated; the mean SNR out of three ROIs within one data set was calculated. Results of the product were compared with every retrospectively reconstructed study data set.

Two radiologists with 8 and 6 years of experience in the field of vascular malformations evaluated the MIPs of the TWIST_3 as well as the product and the iteratively reconstructed TWIST_12 data sets visually in consensus on an imaging workstation. They were blinded to the patients’ medical history and assessed 5 different categories:

### Non-involved interdigital arteries

Delineation of healthy interdigital arteries was assessed in an unaffected finger according to a four-point Likert scale (uniform, nearly uniform, partial delineation, or no delineation).

### Non-involved hand arteries

Healthy, non-involved hand arteries, preferably in the deep palmar arch, were evaluated and graded to a four-point scale (uniform, nearly uniform, partial delineation, no delineation).

### Arterial feeders

Identifiability and delineation of arteries feeding the AVM nidus were evaluated (yes/no).

### Bleeding

Bleeding or blurring artefacts restricting the exact and sharp demarcation of the radial or ulnar artery were assessed on a three-point scale (not present; present, but not diagnostically relevant; present and diagnostically relevant).

### Ghosting artefacts

Multiple reflected, displaced blood vessels (ghosting artefact) were assessed on a three-point scale (not present; present, but not diagnostically relevant; present and diagnostically relevant).

Statistical analysis was performed using the sign-test (IBM SPSS STATISTICS 64-bit MS Windows 23.0.0.0); p-values < 0.05 were regarded statistically significant; results of the TWIST_3 and TWIST_12 data sets were compared with each other as well as every retrospectively reconstructed study data set with the product reconstructed.

### Ethics approval

This was a retrospective single-center consecutive case series. IRB approval was obtained by the Ethikkommission of University of Regensburg, University Medical Center Regensburg, Franz-Josef-Strauss-Allee 11, 93053 Regensburg, Germany; https://ethikkommission.uni-regensburg.de. Individual consent for inclusion in the review was waived by the Research Ethics Board because of the retrospective character of the study. 

We confirm that all methods were carried out in accordance with relevant guidelines and regulations.

### Informed consent

Informed consent for MRI was obtained.

## Results

11 patients (n = 9 females, n = 2 males) with an average age of 43.9 years (min: 27.3 years, max: 78.3 years) were included in the study. MRI data acquisition was successful in all patients. In 1 patient, the iterative reconstruction with a regularization factor of 0.008 was not available.

TWIST_12 MRA of the hand was acquired with 39.8% on average of a single dose gadolinium (20.0–50.0%) corresponding to a total amount of 7.5 mL of contrast medium. Calculation time for the iterative reconstruction took about 8 min 30 s for 50 volumes with a 320 × 320 matrix size.

### Delay

**Figure 1:**
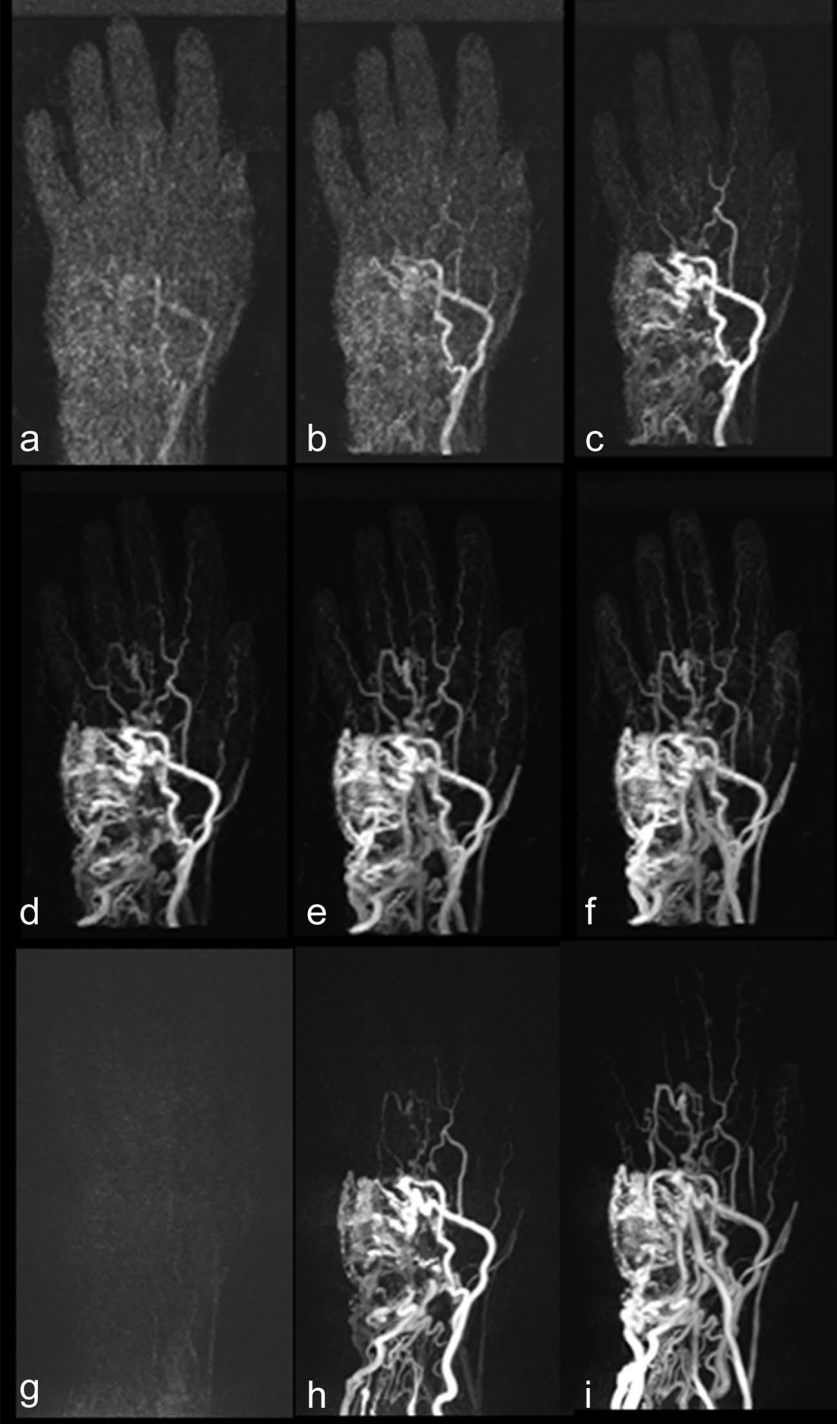
MIPs of successive 3D data sets acquired with TWIST_12 iteratively reconstructed with regularization factor 0.004 (**a**–**f**) and TWIST_3 (**g**–**i**): TWIST_12 with iterative reconstruction (**a**–**f**) improves the differentiation of arterial and venous phase and of arterial feeders.

Concerning the standard TWIST_3 protocol, three patients demonstrated contrast-medium enhancement of major arteries and dominant outflow veins simultaneously on the same MR data set, thus making discrimination impossible. Using TWIST_12 and a regularization factor of 0.004, in all patients a differentiation between major arteries and dominant outflow veins was possible with a gap of 1–9 data sets between arterial and venous phase (Fig. 1).

### SNR

**Figure 2:**
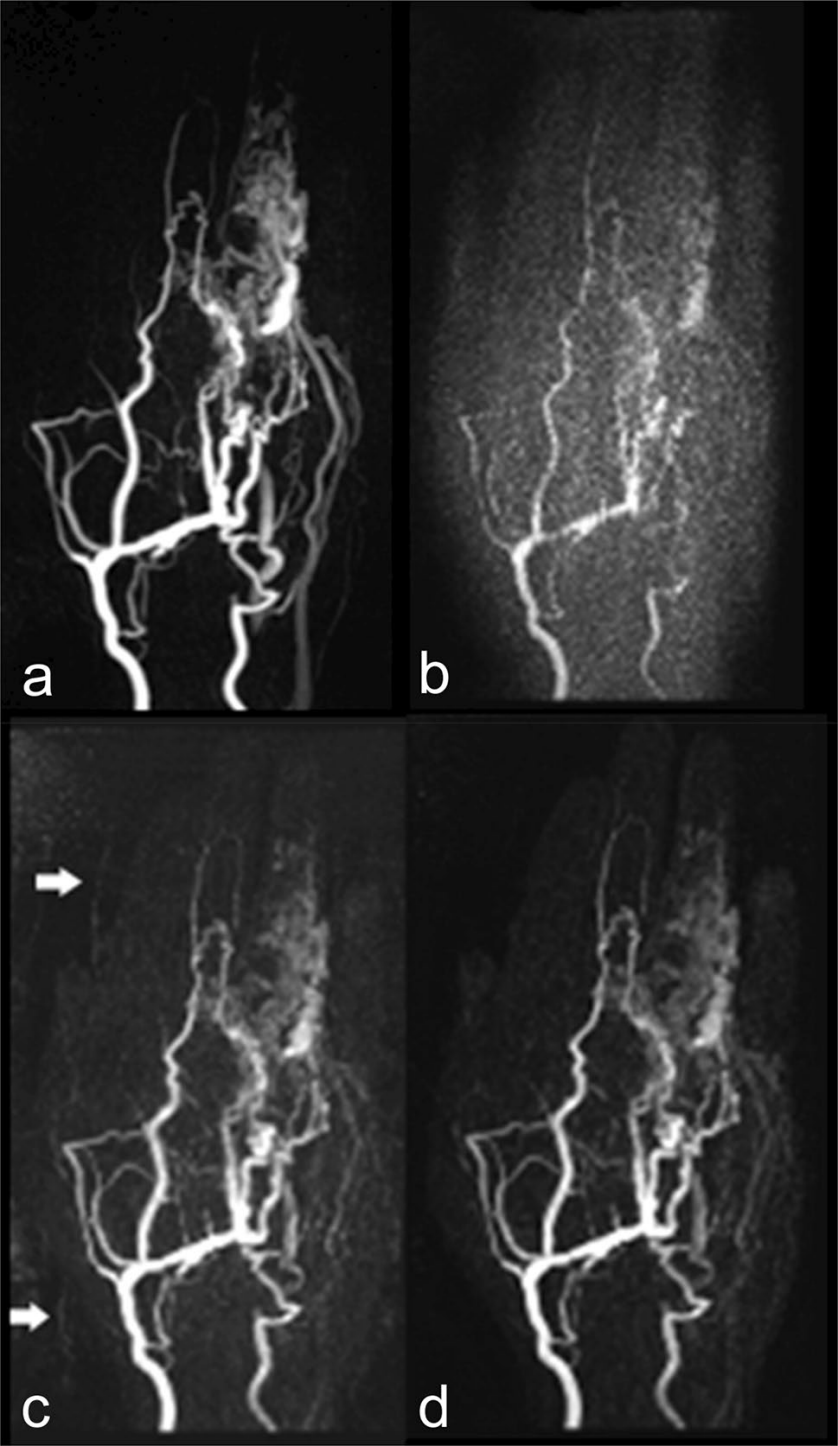
MIPs of arterial phase from TWIST_3 **(a)** and TWIST_12 without **(b)** and with iterative reconstructions (regularization factor 0.001 **(c)**, 0.004 **(d)**): improved SNR after iterative reconstruction **(c, d)**, reduction of ghosting (arrows) by increase of regularization factor **(c **vs.** d)**.

Using iterative reconstructions, SNR was improved by a mean factor of 2.1 (1.9 – 2.3) compared to the TWIST_12 study protocol without iterative reconstructions. SNR improvement declined with increasing regularization factor (Table 1, Fig. 2, ESM Diagram 1).

**Table 1:** Results of quantitative and qualitative evaluation for SNR (signal-to-nose ratio) and the number of patients (in % respectively) with uniform, nearly uniform, partial, or no delineation of non-involved interdigital and hand arteries, with successful or no delineation of arterial feeders, and with differently distinctive bleeding or ghosting artefacts.

	TWIST_3 n = 11	TWIST_12 n = 11	TWIST_12 RF 0.001 n = 11	TWIST_12 RF 0.002 n = 11	TWIST_12 RF 0.004 n = 11	TWIST_12 RF 0.008 n = 10
Mean SNR		12.1	27.3	26.1	24.2	23.1
Improvement of SNR in relation to TWIST_12			2.3	2.2	2.0	1.9
**Delineation of non-involved interdigital finger arteries**						
Uniform	3 27%	5 45%	0 0%	0 0%	1 9%	0 0%
Nearly uniform	6 55%	0 0%	3 27%	6 55%	4 36%	2 20%
Partial	2 18%	5 45%	5 45%	3 27%	3 27%	1 10%
No delineation	0 0%	1 9%	3 27%	2 18%	3 27%	7 70%
**Delineation of non-involved hand arteries**						
Uniform	10 91%	0 0%	7 64%	9 82%	10 91%	9 90%
Nearly uniform	0 0%	4 36%	4 36%	2 18%	1 9%	1 10%
Partial	1 9%	5 45%	0 0%	0 0%	0 0%	0 0%
No delineation	0 0%	2 18%	0 0%	0 0%	0 0%	0 0%
**Arterial feeders**						
Yes	8 73%	8 73%	10 91%	11 100%	11 100%	10 100%
No	3 27%	3 27%	1 9%	0 0%	0 0%	0 0%
**Bleeding**						
Not present	9 82%	10 91%	4 36%	4 36%	6 55%	8 80%
Not relevant	2 18%	1 9%	2 18%	5 45%	5 45%	2 20%
Diagnostically relevant	0 0%	0 0%	5 45%	2 18%	0 0%	0 0%
**Ghosting**						
Not present	11 100%	10 91%	2 18%	2 18%	4 36%	8 80%
Not relevant	0 0%	1 9%	2 18%	2 18%	4 36%	2 20%
Diagnostically relevant	0 0%	0 0%	7 64%	7 64%	3 27%	0 0%

*RF* regularization factor.

### Non-involved interdigital arteries

There was no relevant difference in the delineation of normal finger arteries on TWIST_3 and TWIST_12 protocols (Table 1). Iterative reconstructions did not improve uniform delineation of healthy finger arteries. On the contrary, after iterative reconstruction with the regularization factors 0.001 and 0.008, results worsened significantly (p = 0.039); whereas regularization factors 0.002 (p = 0.18) and 0.004 (p = 0.289) led to a statistically not significant deterioration of results.

### Non-involved hand arteries

Using the standard TWIST_3, uniform delineation of healthy hand arteries was possible in ten patients. Whereas on TWIST_12 images without iterative reconstruction the delineation of healthy hand arteries declined statistically significantly (p = 0.001) (Table 1), post-processing data with all regularization factors improved these results significantly (regularization factors 0.001 and 0.008: p = 0.002; regularization factors 0.002 and 0.004: p = 0.001).

### Arterial feeders

Arterial feeders were detectable in eight patients on TWIST_3 and on TWIST_12 images, respectively. Iterative reconstructions with all regularization factors improved the detectability of arterial feeders in comparison to TWIST_12, but results did not differ significantly (regularization factors 0.002, 0.004, 0.008: p = 0.25; regularization factor 0.001: p = 0.625). The regularization factors 0.002, 0.004, and 0.008 enabled the detection of arterial feeders in all patients (Table 1, Fig. 1).

### Bleeding

On TWIST_3 and TWIST_12, no diagnostically relevant bleeding artefact was diagnosed. Results worsened significantly with the regularization factors 0.001 (p = 0.016) and 0.002 (p = 0.031). Increasing the regularization factor helped to extinguish those disadvantages (regularization factor 0.004: p = 0.125; regularization factor 0.008: p = 1.00) (Table 1).

### Ghosting artefacts

On TWIST_3 and TWIST_12 MRA, no diagnostically relevant ghosting artefacts were present. With low regularization factors (0.001 and 0.002), ghosting artefacts deteriorated significantly (p = 0.008). Higher regularization factors improved ghosting artefacts (regularization factor 0.004: p = 0.07; regularization factor 0.008: p = 1.00) (Fig. 2, Table 1).

## Discussion

Conventional DSA today still presents the gold standard for the imaging of AVMs^11^. However, this technique has the disadvantages of invasiveness, radiation dose, and iodinated contrast-medium^10^. MRA represents an alternative non-invasive imaging modality, but the exact delineation of subtle hemodynamic and anatomical detail of AVMs is still challenging^12^. Two different aspects have to be considered: morphologic information—in terms of a high spatial resolution—is important for three-dimensional understanding of AVMs and thus for treatment planning. Furthermore, the exact analysis of the hemodynamics is crucial^12^. As AVMs are high-flow malformations consisting of multiple small direct shunts between arteries and veins, a high temporal resolution is mandatory for the understanding of blood flow. Furthermore, it is important to delineate the blood supply of the nidus as well as of tissue located distal to the nidus in order to embolize the nidus without the risk of distal tissue necrosis and potential amputation^6^. To date, the benefit of TWIST MRA and parallel MR imaging techniques has been studied for information regarding morphologic and hemodynamic parameters of the extracranial carotid circulation. Acquisition speed increased generally, but artefacts restricted the results^10^.

A well-known disadvantage of these very fast sequences is the loss of signal, resulting in a low SNR as well as blurring of small vessels ^18^. Wetzl et al. have shown advantages of iterative reconstructions especially in improving the delineation of small vessels of the lung^15^.

In our institution, a TWIST_3 standard protocol with a 3D data set reconstructed every 5.57 s was established for treatment planning and post-therapeutically imaging of AVMs. Whereas morphologic information was deemed to be adequate in the clinical routine, the temporal resolution with a data set every 5.57 s constituted a lack of information regarding the exact differentiation of arteries from veins in this fast flow situation.

For optimizing the temporal resolution, we set up a very fast TWIST_12 study protocol depicting data sets every 1.44 s and examined AVMs of the hand^19^. Thus, we wanted to study the method´s reliability and robustness in preferably small vessels. Furthermore, we investigated the additional value of different iterative reconstructions using four different regularization factors. Comparison between reconstructed sequences with the product TWIST_12 reconstruction was performed.

To the best of our knowledge, this is the first study to examine the effects, advantages, and disadvantages of rapid sequences with iterative reconstructions with different regularization factors on AVMs of the hand.

As we had hypothesized, the major advantage of the study protocol was the high temporal resolution enabling to differentiate between arteries and veins in all patients.

On TWIST_12 data sets, diagnostically relevant artefacts were absent. Non-involved interdigital arteries were uniformly definable in 45% of patients in comparison to 27% of patients on TWIST_3 standard images. Disadvantages of the study protocol were the low SNR, the lack of uniform delineation of non-involved hand arteries, and the detectability of arterial feeders in only 73% of patients.

Whereas the regularization factors 0.001 and 0.002 improved the SNR, the former one maximally, diagnostically relevant artefacts were significantly more frequent in comparison to TWIST_12 (bleeding: p = 0.016 and p = 0.031, ghosting: p = 0.008 and p = 0.008 respectively).

The regularization factor of 0.004 led to a less distinct improvement of SNR compared to iterative reconstructions with the factor 0.001. The uniform delineation of non-involved hand arteries improved significantly (p = 0.001) in comparison to the unmodified TWIST_12, arterial feeders were detectable in all patients, and diagnostically relevant artefacts decreased in comparison to iterative reconstruction with lower regularization factors.

The regularization factor of 0.008 improved the SNR in comparison to the study protocol, but to a lesser degree than all other regularization factors. Non-involved hand arteries (p = 0.002) and arterial feeders were detectable at a high rate. Furthermore, diagnostically relevant artefacts were extinguished.

Contrary to the results of other study groups, where the delineation of very small vessels improved after iterative reconstructions^15^, the delineation of non-involved interdigital arteries worsened with higher regularization factors resulting in a lack of its delineation in 70% of patients after reconstructing with a factor of 0.008.

Considering the uniform and nearly uniform delineation of non-involved interdigital arteries as diagnostically accurate, the best result was TWIST_3 in 82% of patients succeeded by TWIST_12 with regularization factor 0.002 (55%), and TWIST_12 with product reconstruction (45%).

Thus, we sub-classified all assessed findings into diagnostically accurate and into non-diagnostic categories and summarized results. Regarding the delineation of non-involved interdigital and hand arteries, the uniform and the nearly uniform depiction was regarded as diagnostically accurate. Furthermore, the identification of arterial feeders and the absence of relevant artefacts were deemed as diagnostically accurate. Results of diagnostically accurate results are displayed in ESM Diagram 2.

The best overall results were achieved with the regularization factors 0.004 and 0.008. Whereas the factor 0.008 extinguished all artefacts completely, it had the disadvantage of a lesser SNR and a not accurate delineation of non-involved interdigital arteries. Iterative reconstructions with regularization factor 0.004 outperformed the factor 0.008 in both categories on account of a higher rate of artefacts.

The results in this study were achieved with an average dose of 39.8% of a single dose of contrast medium only. Thus, iterative reconstructions might help to reduce the required contrast medium amount. Taking into account the detection of gadolinium deposition in the brain after repetitive gadolinium applications for MRI, a low dose of gadolinium has to be achieved, especially in this young patient group facing—due to their chronic disease—further contrast-medium injections for MRI^20^.

Despite the retrospective character of this study, results are promising for accurate imaging and delineation of AVM of the hand and might also be transferred for imaging of AVM of the feet. However, further investigations in a larger study population is necessary.

In conclusion, a high temporal and spatial resolution is mandatory for delineation and understanding of hemodynamic features in AVMs. Temporal resolution is sufficient using an extraordinarily high acceleration factor of 12, but is associated with decrease in SNR. The postprocessing with iterative reconstructions improves the SNR and results in diagnostically reliable data sets, and reduces volume of applied contrast medium.

## Supplementary information

Supplementary Information.

## Data Availability

All data is included in the manuscript in terms of Table 1.
