# Supplementary material for: Fast TWIST with iterative reconstruction improves diagnostic accuracy of AVM of the hand
Source: Sci Rep. 2020 Oct 1;10:16355. doi: 10.1038/s41598-020-73331-6 (PMC7529883; doi:10.1038/s41598-020-73331-6)
Supplement: Supplementary file 1 — Supplementary Information. [file 41598_2020_73331_MOESM1_ESM.docx]

**Fast TWIST with iterative reconstruction improves diagnostic accuracy of AVM of the hand.**

Corresponding author:

**Veronika I. Huf ***

veronika.huf@ukr.de, Department of Radiology, University Medical Center Regensburg, 93042 Regensburg, Germany

**Claudia Fellner**

claudia.fellner@klinik.uni-regensburg.de, Department of Radiology, University Medical Center Regensburg, 93042 Regensburg, Germany

**Walter A. Wohlgemuth**

walter.wohlgemuth@uk-halle.de, Interdisciplinary Center for Vascular Anomalies, University Clinic and Polyclinic of Radiology, University Hospital Halle, 06120 Halle (Saale), Germany

**Christian Stroszczynski**

christian.stroszczynski@klinik.uni-regensburg.de, Department of Radiology, University Medical Center Regensburg, 93042 Regensburg, Germany

**Michaela Schmidt**

michaela.schmidt@siemens-healthineers.com**,** Siemens Healthcare, 91052 Erlangen, Germany

**Christoph Forman**

christoph.forman@siemens-healthineers.com, Siemens Healthcare, 91052 Erlangen, Germany

**Jens Wetzl**

jens.wetzl@siemens-healthineers.com, Siemens Healthcare, 91052 Erlangen, Germany

**Wibke Uller**

wibke.uller@klinik.uni-regensburg.de, Department of Radiology, University Medical Center Regensburg, 93042 Regensburg, Germany

**Diagram 1:** Mean signal-to-noise ratio (SNR) for all 5 study data sets

RF: regularization factor

**Diagram 2:** Percentage of patients with diagnostically accurate results for

each study data set in the assessed categories

RF = regularization factor
